# LearnOvation: an intervention to foster exploration and exploitation behaviour in health care management in daily practice

**DOI:** 10.1186/s12913-019-4152-8

**Published:** 2019-05-22

**Authors:** Gunilla Avby, Sofia Kjellström

**Affiliations:** The Jönköping Academy for Improvement of Health and Welfare, School of Health and Welfare, Jönköping University, P.0. 1056, S-55111 Jönköping, Sweden

**Keywords:** Exploration, Exploitation, Health care research, Experimental cross-over design

## Abstract

**Background:**

Innovation has been identified as an important engine for improving the quality, productivity and efficiency of health care. Little is known about how to stimulate innovation capacity in primary health care in general; even less is known about how specific interventions should be designed to support managements’ work with practice-based innovations. Research has shown that if managers and teams are excellent at handling the challenges of production (exploitation) and development (exploration), they are better at innovation. The aim of the study is to develop a dynamic management support programme to increase innovation leadership skills in daily practice.

**Methods:**

The study has an interactive approach that allows the need for empirical and theoretical knowledge to emerge and merge, and a quasi-experimental cross-over design. Eight primary health care centres will participate in the study. In the first phase, the management teams at four health care centres will participate in the intervention, and the other four centres will serve as a control group. Thereafter, the units will switch places and the control group will experience the intervention. All staff at the 8 units will answer questionnaires at four points in time (before, during, after, 6 months later) to evaluate the effects of the intervention.

**Discussion:**

The study will contribute to knowledge on how to organize processes of innovation and support exploitation and exploration behaviours by co-producing and testing a tailor-made management support programme for innovation work in primary health care. An expected long-term effect is that the support system will be disseminated to other centres both within and beyond the participating organizations.

**Electronic supplementary material:**

The online version of this article (10.1186/s12913-019-4152-8) contains supplementary material, which is available to authorized users.

## Background

Innovation is proposed to improve the quality, productivity and efficiency of health care [1, 2]. The importance of health care providers focussing on support systems for innovations in daily practice has been stressed as a response to growing public demands and political pressures [3, 4]. Research has shown that there is a strong link between innovation and leadership in the public sector [5–10], but relatively few studies have aimed at understanding how professionals themselves prompt change [11].

Managers can create a supportive culture for bottom-up innovations by different means, such as consulting staff, promoting innovators, and protecting them from control-oriented central agencies [7]. Furthermore, managers can enhance innovations by handling the need to produce (exploitation) and the need to develop (exploration) [12, 13] and finding an appropriate balance between the two quite different activities. Although innovation in the public sector is a relatively new field [14–16], the quality improvement paradigm has been a forerunner and parallel management model of public sector innovation [17, 18]. Studies show that innovations are supported by a strong culture and climate of learning and collaboration [19], and a receptive capacity for new knowledge [20]. Most innovations are actually outcomes of combinations; that is, integration of diverse forms of knowledge and interaction when people with different backgrounds meet [16, 21].

### Innovation

In this study, innovation is viewed as a function of learning and knowledge creation, integrated into daily work practices, experiences and professional skills, as is the creation or discovery of new solutions, new approaches, or new ideas [22]. Innovation is the introduction of new elements into public service, representing a discontinuity with the past [23]. Innovation is an important form of everyday practice-based learning [24]. With this approach, innovation processes are conceptualized as learning processes and tend to stress not only external drivers of change but also a more intrinsic understanding of innovation. Innovation or learning may thus take its starting point in a disruption of practice and in the practitioner’s uncertainty of how to solve a problem [25, 26]. An uncertainty requires new knowledge, and new knowledge may challenge the boundaries of local practice and collegial cooperation (habits) and as such offers a learning opportunity and basis for innovation. Thus, all learning in work is to some extent innovative in that it introduces change [24], which also makes learning a key concept in research on innovation [27].

Different organizations provide widely different contexts for innovations. Research has found that a learning-promoting climate and culture to mobilize human resources, and a pro-innovation attitude in the organization, are important drivers of change [2, 3, 7, 10, 28]. Rosen [3] suggests that time and resources dedicated to fostering a learning environment are likely to be well invested, as is a greater focus on tangible customers’ needs. However, little is known about how innovation capacity in primary health care in general can be stimulated; even less is known about how specific interventions should be designed to support managers’ work with practice-based innovations.

The key resource in many public services, such as primary care, is undoubtedly the staff’s expertise and capacity for problem solving [29, 30]. The demand for professionals to continually engage in learning and the renewal of professional capacity has been reinforced, and opportunities for reflexive awareness of the impact of informal work processes are considered necessary for promoting and supporting developments in practice [3, 31, 32]. Thus, change must be accompanied by support structures and managerial actions, encouraging new behaviour and facilitating meaning making [2, 11, 33].

Three previous studies of well-functioning primary health care centres in Sweden (in the same region in which this study takes place) showed that professionals were provided with time and space for quality improvement work and that they worked to coordinate their efforts through non-hierarchical relationships and collaborative teamwork [19, 34, 35]. The studies established that professional autonomy was highly valued, sustained and maintained through delegation of responsibility, trust, support and feedback; and that healthy work environments, such as accessible and fair leaders, skilled communication, collaboration/teamwork, employee involvement, and good relations with stakeholders, contributed to the development of a well-functioning health care centre. Conditions that provided a fertile ground for innovations were a culture and climate of managing learning, combined with the ability to monitor performance, adapt to external requirements, and collaborate with others [19].

### Ambidexterity theory and leadership for innovation

The theoretical framework for the intervention in focus is organizational ambidexterity theory. Organizational ambidexterity encompasses the search for the appropriate balance between exploiting existing competencies (the need to produce) and exploring new opportunities (the need to innovate) [13, 36]. Exploitation and exploration are different activities and require quite different abilities within an organization. Whereas exploitation is about using existing knowledge, skills and processes to safeguard customer satisfaction, maintain business as usual and efficiency, exploration concerns creating new knowledge, skills and processes. Thus, when it comes to exploration, organizations must use a creative and dynamic approach to enable innovations and be willing to adjust and change services, products, processes and markets. Exploration concerns transformation, that is, making changes within the organization with the aim of doing something different. It involves risk taking and experimentation.

Within organizations, there is a tension between the need to produce (exploitation) and the need to innovate (exploration). This tension may be met by flexibility and a combination of different leadership behaviours to switch between these two activities, a so-called ambidextrous leadership [37]. Dexterous originates from the Latin word dexter, meaning “on the right side”, and the prefix ambi- means “both”. Thus, ambidextrous describes someone who is equally skilful at using both hands.

Studies show that in organizations where managers have the ability to work with both activities, innovations take place to a greater extent among employees. In the organizations where there is a high degree of both exploitation and exploration, a high degree of innovation also occurs; but less innovation occurs when only exploration is high [38, 39]. Recently, researchers have argued that leaders also need to engage in the tension by providing enabling leadership that bridges the tension between exploitation and exploration activities by creating an adaptive space where new ideas can be scaled within the formal system [40, 41].

The implication of this work is that, on an organizational level, there is a need to select and reward leaders who express both kinds of behaviours [39]. Leaders should be made aware of the importance of both opening and closing behaviours to stimulate knowledge development and knowledge use among employees to simulate innovation [38, 39]. There is a need to raise awareness of the complexity of the innovation process and design education activities that present and practice different elements of ambidextrous leadership. These include exploration behaviours, such as allowing errors and experimentation, and exploitation behaviours, such as monitoring and controlling, allowing routines, and keeping plans. Similarly, employees should learn about experimentation and exploitation [39]. There is a risk of not putting emphasis on exploitation when innovation work is initiated or that that aspect is neglected [38, 39].

## Methods

### Aim and research question

The aim of the study is to develop a dynamic management support programme to increase managements’ innovation leadership skills in daily practice. The intervention that is the keystone of the study will be based on introducing and practicing exploration and exploitation behaviours. The intervention is theoretically based on current research and tailor made to meet the learning needs of primary health care managers working in Jönköping County.

After the intervention:

- The managers will have insight and tools to support and carry out innovations at the workplace.
- The professionals will have knowledge on innovation processes and have increased their use of general knowledge in daily practice.
- The managers and staff will be equipped to realize innovations in daily practice.

The study addresses the research question: How can primary health care concurrently satisfy the need to innovate while fulfilling the everyday demands of existing patients, owners and regulations?

### Setting

Sweden has a decentralized health care system, and primary health care centres employ a multidisciplinary workforce, which in a global comparison tends to be quite unusual, although there are similar systems in Finland, the United Kingdom, and the Netherlands [42]. A common setting is 4–10 physicians specialized in general practice, working together with other health care professionals, such as nurses, specialist nurses, physiotherapists, occupational therapists, social workers and cognitive therapists [42].

The local setting for this study is primary care in southern Sweden. The study is an expansion of an ongoing collaboration between three partners: Jönköping University, Bräcke diakoni (BD), and Region Jönköping County (RJC). Also, the study has its starting point in a framework that was developed in a previous study on innovations at successful primary health care centres in Jönköping County [19]. The collaborating health care organizations have a long track record and experience of similar development strategies based on continuous quality improvement in everyday work combined with a strong value base focused on the needs of patients [35].

### Design

The study has an interactive approach that allows the need for empirical and theoretical knowledge to emerge and merge, producing knowledge of practical relevance and a high scientific standard. Interactive research emphasizes joint learning between the participants and the researchers throughout the research process [43, 44]. More specifically, close collaboration between practice and research allows for common interpretation and exploration of findings, in addition to extensive sharing of knowledge. Such an approach has proven successful in workplace research and feasible in creating conditions for developmental work in practice [44].

**Fig. 1:**
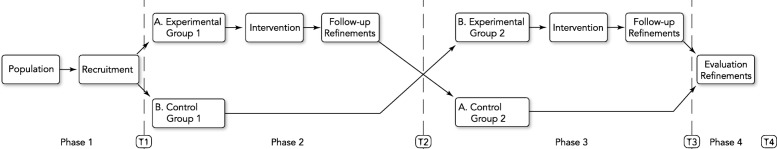
Design of the intervention

A quasi-experimental cross-over design will be used (see Fig. 1).

1. In the first phase, 8–10 health care centres will be recruited. This phase has been completed.
2. The recruited centres will then be divided into two groups (i.e. intervention groups): A, experimental group 1 will participate in the first intervention; B, control group 1 as the control group for the first intervention.
3. When the first intervention is completed, groups A and B will switch places and experimental group 1 will become control group 2 and control group 1 will become experimental group 2.
4. The fourth phase concludes in a follow-up activity, and necessary refinements will be made to the intervention for future implementation.

### Hypothesis

Three hypotheses will be tested in the intervention:

- Hypothesis 1: Innovations in daily practice increase when managers encourage employees’ use of knowledge.
- Hypothesis 2: The management team creates better conditions for innovation processes in daily practice after having participated in the intervention.
- Hypothesis 3: The professional’s innovativeness increases after the intervention.

### Intervention groups

In total, eight health care centres have been recruited and divided into two intervention groups (i.e. A and B). Each intervention group includes four health care centres: two from RJC and two from BD. The manager and an additional 2–3 persons from the management team in each centre will participate in the study.

### Recruitment process

The recruitment process was different in the two organizations. In BD, the care centres were selected by the R&D unit based on size and management prerequisites. In RJC, the recruitment process was initiated by sending out general information about the opportunity to participate in the study to all care centres. The centres were given further information about the study at a management conference, where they also were encouraged by one of the researchers to sign up for the intervention. This process resulted in one care centre volunteering to participate. A more active stance was taken, and three additional centres were recruited by personal communication.

### Development of the intervention

The aim is to tailor the intervention to meet the learning needs of primary health care management teams working in Jönköping County, delivered within the context of their work environment. The intervention was created in several steps: (1) needs analysis, (2) feedback and consolidation, (3) development and implementation of the intervention.

1. In order to create a needs analysis, interviews were performed at the participating organizations with health care managers (*n* = 6) and a selection of professionals working with innovation (*n* = 5). The following questions were addressed in the first step: What needs and what challenges are related to the organizations’ capacity for innovation? What support and steering is needed in the organizations to realize, implement and sustain innovations? To create a broader picture of what is needed to increase an organization’s innovativeness, the researchers investigated previous and similar innovation interventions in Sweden.
2. The data collected in the first step were presented to the participating organizations, and they were asked to select a few representatives who could serve as a steering group for the study. Once these representatives were allocated, a series of joint partnership meetings were conducted to analyse the data and identify the most important needs to increase the organizations’ innovativeness. An important aspect was to align the intervention with existing development activities to avoid parallel tracks. Thus, the members of the steering group were asked to map and present ongoing activities at their organizations. Also, the members tested and verified the needs identified with their colleagues. In this step, the following question is addressed: What do the local processes for knowledge use and knowledge development look like and how may the intervention align with these?
3. Enriched with the results from steps 1 and 2, the intervention was crafted and structured based on the four preconditions identified in Avby et al. [19]. At this stage, the intervention will also be tested, which will greatly increase the activity at the participating health care centres and involve the entire staff. The participants will be assigned tasks to perform with their staff.

### Intervention content and structure

The intervention will consist of four half-day learning seminars, ending with lunch together. The four overarching themes are derived from a framework developed in a previous study [19]: (1) managing learning; (2) monitoring performance; (3) adjusting to requirement; and (4) collaborating with others. Each theme also relates to explore and exploit aspects which are measured through four questionnaires (see Table 1). The first seminar will cover knowledge about exploring and exploiting behaviours, present results from a pre-questionnaire completed at the participating health care centres, and address current research on well-functioning primary health care centres. The second learning seminar will encompass theoretical and practical knowledge of how to monitor performance and how to work systematically with quality improvement work, measurements, and action plans. The third seminar will include the theory in use and espoused theory and how to provide feedback. The final seminar will focus on activities directed at learning more about co-production and teamwork.

**Table 1:** Intervention themes related to items items measured through four questionnaires

Questionnaire items	Learning seminar 1	Learning seminar 2	Learning seminar 3	Learning seminar 4
Avby et al., [19]	Managing learning	Monitoring performance	Adjusting to requirements	Collaborating with others
Rosing et al., [38]	Allows different ways of accomplishing a task	Monitors and controls goal attainment	Sanctions errors	
	Encourages experimentation with different ideas	Sticks to plans	Controls adherence to rules	
	Motives to take risks	Established routines		
	Gives possibilities for independent thinking and acting	Takes corrective action	Pays attention to uniform task accomplishment	
	Gives room for own ideas			
	Allows errors			
	Encourages error learning			
Mom et al., [45]	Searching for new possibilities with respect to products/ services, processes or markets	Activities of which a lot of experience has been accumulated by yourself	Activities requiring quite some adaptability of you	
	Evaluating diverse options with respect to products/ services, processes or markets	Focusing on strong renewal of products/ services or processes	Activities which serve existing (internal) customers with existing services/ products	
	Activities requiring you to learn new skills or knowledge	Activities primarily focused on achieving short-term goals	Activities of which it is clear to you how to conduct them	
			Activities which you can properly conduct by using your present knowledge	
			Activities which clearly fit into existing company policy	
Welbourne et al., [46]	Coming up with new ideas	Working to implement new ideas		
	Finding improved ways to do things	Creating better processes and routines		
New questions for this study				Patients and users
				My own profession
				Other professional groups at the primary health care center
				Coworkers and managers
				Students
				Other primary health care centers
				Universities and colleges
				Other stakeholders

Every learning seminar will combine theoretical input, reflection and practical exercises. Between the seminars, each management team will be expected to carry out a set of actions at home that supports their innovation efforts (progress). Because primary health care is under a lot of pressure, time will be allocated at the seminars to prepare these home activities, and sessions will be offered for the participants to share experiences and tips on how to progress.

The intervention will engage a mix of researchers and practitioners who will have slightly different roles and responsibilities. The researchers will address the different themes with up-to-date research and support a meta-reflection on the issues discussed (abstract and general knowledge), and the practitioners will provide in-depth knowledge from lengthy experience of working with quality improvement work (practice-based and local knowledge).

### Pedagogical approach

A basic understanding behind this study is that human capital is the fundamental input of change. Individual behaviour is always performed within contextual constraints. Ultimately, the key to innovation is the professional’s engagement in the work, that is, their willingness, capability and opportunity to innovate [19]. Research shows that management and leaders, by developing a culture and climate of learning, can support an innovative friendly work environment [3, 5, 28].

How, then, does learning at work lead to innovation? Basically, working, learning and innovating are closely related forms of human activity. However, learning only from experience is not sufficient in handling the growing complexity in work and the demands of different stakeholders, because experience often gives us little information to learn from. Interventions developed with the aim of introducing new knowledge, such as research-based knowledge, and are relevant to address an actual practical problem might challenge and critically explore well-established attitudes, beliefs, and practices [19, 47]. Thus, reflection is an important activity that needs to be addressed. Reflection helps to translate experiences into learning by examining one’s attitudes, beliefs and actions, to draw conclusions to enable better choices or responses in the future [26]. Also, a better insight in how to use reminders and vigilant monitoring is useful for disrupting habits, and thus offers learning opportunities [48].

Innovation work is a relatively new phenomenon in the public sector, which makes it difficult to embrace in the prevailing management and professional logic. Many consider innovation as a new question that needs to be addressed in organizational goals, strategies and visions. Thus, management’s understanding of the phenomenon is necessary. Our strategy is to incorporate theoretical input on public sector innovation and organizational prerequisites for practice-driven innovation in the learning seminaries to develop the management team’s know-how to develop a fertile ground for exploration and exploitation and provide different tools that support and structure innovation work. Keywords are knowledge development, reflection and practice.

### Evidenced-based practice in the design of the intervention

The overall design of the intervention has its roots in evidenced-based practice, by combining different knowledge sources [49]. Furthermore, the intervention is based on a needs analysis, which has been argued results in greater transfer and learning [50]. Programmes that are both evidence-based and practically relevant, namely created and conducted with practitioners and academic partners, have been found to have more effective outcomes [51]. Also, unique to this intervention is the constant presence and reflections of the participant’s requirements to enable innovations. Throughout the intervention, the participants will identify their particular needs and tools will be provided for them to try out regarding their particular needs.

Research suggests that the training design (intervention) should align with and be based on desired outcomes, such as organizational results, transfer, and learning [50, 51]. Leadership training programmes tend to be more effective for cognitive learning, cognitive transfer, skill-based learning, and skill-based transfer than affective learning and affective transfer [51]. In this intervention, we have included (and evaluated) cognitive and/or skill-based content. The intervention is based on the skills needed for ambidexterity, thus exploring and exploiting behaviours will be introduced at the first learning seminar.

Delivery methods influence outcomes, and the most effective programmes are suggested to have a mix of methods [50, 51]. Our intervention will use multiple delivery methods with various learning activities: information (i.e. short lectures, discussion, study material), demonstration (e.g. reflective exercises), and practice (i.e. on-the-job training exercises). Practice has been found to be the most important dimension, which has encouraged us to incorporate practical tasks in the seminars, between the seminaries and follow-up discussions. An important base is for the training (intervention) to include experiencing and engaging in the practices that support innovation while learning about them [27, 52]. To give the participants first-hand experience of activities that they can do at their own unit, teachers must model reflective practices, skills, and abilities in the intervention.

We have also included elements of feedback throughout the intervention, because feedback is an essential aspect that enhances learning and needs to be adapted [22, 24, 26, 31, 32].

Evidenced-based leadership training provides multiple training sessions separated by time rather than a single training session [51]. Our intervention will consist of four sessions a 4-week intervals.

At the first session, and throughout the intervention, we will foster psychological safety. Psychological safety creates a space where members can openly discuss errors without fear of punishment, which is critical in team training [53]. Learning from errors has been shown to be facilitative.

### Before and after questionnaires for managers and staff

The effects of the intervention will be evaluated through questionnaires distributed to managers and staff at the primary health care centres at four times in a before and after design. All managers and staff will answer a questionnaire at four points in time: (1) T1, before; in phase 1 before the first intervention starts; (2) T2, after intervention 1; after concluding phase 2; (3) T3, after intervention 2; after concluding phase 3; and (4) T4, 6 months after the intervention (see Fig. 1).

The work on the baseline questionnaire is underway. The managers were sent an e-mail, including an attachment with information about the study and ethical considerations to be forwarded to all staff. The questionnaire was then distributed in paper format at a staff meeting. Absent staff were given the questionnaire in person or it was sent by e-mail. At each centre, a key person has been assigned to collect the questionnaires and be responsible thereafter for forwarding the questionnaires to one of the researchers, who will finally code and register the results.

The questionnaire consists of four constructs: (1) explorative and exploitive behaviours (EEB) [45] has 12 items rated on a scale of 1 to 5; (2) opening and closing behaviour (OCB) [36] has 14 items rated on a scale of 1 to 5; (3) employee self-reported innovative performance [46] has four items rated on a scale of 1 to 5; and (4) collaboration within and outside the unit is a newly constructed measurement for this study. A strength of the questionnaire is that the items are clearly related to the themes and content of the intervention, which means that the proper outcomes of the intervention is measured by the questionaries (see Table 1). Constructs 1–3 have been translated into Swedish and piloted in a validation process, where practitioners and Masters students of Quality Improvement and Leadership have provided feedback. The fourth construct was originally in Swedish.

1. Explorative and exploitive behaviours (EEB). This questionnaire was originally used for leaders to assess their own behaviours [45]. In later studies, employees self-reported on their exploitation and exploration behaviours (6 + 6 items) rated on a scale of 1 to 5. In a later study, an item was excluded because it did not work so well in a German translation [39].
2. Opening and closing behaviour (OCB). In this questionnaire, the employees assess a leader’s behaviour. Items are based on exploitation and exploration theory [36, 38]. Behaviours are rated according to open (explore) or closed (exploit) character (7 + 7 items). The items are rated on a scale of 1 to 5.
3. Employee self-reported innovative performance. This is a self-reported assessment of how innovative a person is, whereby the participant assesses four statements that form a sub-scale of innovative behaviour (creativity and innovation in one’s job and the organization as a whole) within the framework of a more comprehensive instrument of employee performance (Role Based Performance Scale) [46]. There are five items: coming up with new ideas, working to implement new ideas, finding improved ways to do things, creating better processes and routines. The items are rated a 5-point scale from 1 (needs much improvement) to 5 (excellent).
4. Internal and external collaboration. This newly created questionnaire consists of three items about collaboration within a unit, within a profession, between different professional groups, and between employees and managers (see Additional file 1). There are two items about collaboration across units, about collaboration across professionals/professional groups, and between units and management functions. The items are rated on a 5-point scale from 1 (never) to 5 (always).

### Data analysis and power

Quantitative data in the questionnaires will be analysed with descriptive analyses (e.g. frequencies, means and correlations), but also using more advanced methods such as multi-level modelling and structural equation modelling [39], which are used to analyse between cases and over time. The three translated questionnaires will be tested psychometrically by Rasch analyses, which includes factor analysis and analysis of the items.

A population of 128 participants (64 per group) is needed for 80% power with a significance level of 0.05. It is reasonable to merge the intervention groups from rounds 1 and 2, and the control groups in the same way, because the total number of respondents involves four primary care centres, with responses from at least 20 employees for rounds 1 and 2, giving 160 potential respondents in total. Because all respondents will be in both a control group and an intervention group, the measurements before and after the intervention can be compared, giving a maximum of 160 possible respondents in each group. But in the comparison between intervention group 1 and control group 1, there will be approximately 80 respondents per group. Realistically, a response rate of 60–80% may be possible, which would mean 48–64 respondents, which would give low but reasonable power because we have approached all possible participants. When merging the total group, you also have the opportunity to measure the difference between before and 6 months after measurements and then have a reasonable opportunity to get 64 respondents of the 160 potential respondents both before and after (40% respondents).

## Discussion

The project is expected to contribute to experiences and knowledge on how to organize for processes of innovation. The project is also expected to provide a management support system for innovation work that has been implemented at participating primary health care centres. A long-term effect is anticipated to be that the support system will be disseminated to organizations both within and outside health care. The project will contribute to improvement science by providing an evidenced-based intervention that is concurrently based on ambidexterity theory and tailor made for the needs of primary care; it will also be adapted during the process to fit the needs even more. The intervention is not based on the notion that the homework for all centres will be the same; each unit will work on the issues that are most important for its particular needs.

Many things affect the results of an intervention, but the character and challenges of the intervention groups are major factors. The original idea was that primary care centres should be matched in order to create a good scientific design that was based on the presumption that we had a pool of primary care centres to choose from. That was not the case, one of the main reasons being that primary care centres are under pressure. We had problems recruiting members at the start. However, we know that all planned participants have identified needs for improving innovation work.

We hope the intervention will be appreciated as creating value for the participating units. We have also recruited two (three+) of the most successful units in the region to answer the questionnaire at T0. Their results will be used as a measure of the baseline or best practice values. This will further add to the practical value of the intervention for the participating primary health care centres and add scientific value to the field of implementation sciences. To ensure the quality of the intervention, the TIDieR (Template for Intervention Description and Replication) has been used [54].

## Additional file

Additional file 1: Internal and external collaboration questionnaire, A questionnaire created for the LearnOvation intervention. (DOCX 21 kb)

## Abbreviations

BD: Bräcke diakoni
EEB: Explorative and exploitive behaviours
OCB: Opening and closing behaviour
RJC: Region Jönköping County
