# Supplementary material for: LearnOvation: an intervention to foster exploration and exploitation behaviour in health care management in daily practice
Source: BMC Health Serv Res. 2019 May 22;19:319. doi: 10.1186/s12913-019-4152-8 (PMC6529990; doi:10.1186/s12913-019-4152-8)
Supplement: Supplementary file 1 — Internal and external collaboration questionnaire, A questionnaire created for the LearnOvation intervention. (DOCX 21 kb) [file 12913_2019_4152_MOESM1_ESM.docx]

Internal and external collaboration questionnaire

Assess your own primary health care center´s ability to collaborate (the last 2 months), on a 5-point scale from 1 (never) to 5 (always).

1= Never

2 =Seldom

3 = Sometimes

4 = Often

5 = Always

We cherish and develop ideas with…

- Patients and users
- My own profession
- Other professional groups at the primary health care center
- Coworkers and managers
- Students
- Other primary health care centers
- Universities and colleges
- Other stakeholders
